# The impacts of the COVID-19 pandemic on treatment-resistant schizophrenia patients having followed virtual reality therapy or cognitive behavioural therapy: a content analysis

**DOI:** 10.1080/07853890.2022.2121852

**Published:** 2022-09-14

**Authors:** Alexandre Hudon, Nayla Léveillé, Katerina Sanchez-Schicharew, Laura Dellazizzo, Kingsada Phraxayavong, Alexandre Dumais

**Affiliations:** a Centre de Recherche de l‘Institut Universitaire en Santé Mentale de Montréal, Montreal, Canada; b Department of Psychiatry and Addictology, Faculty of Medicine, Université de Montréal, Montreal, Canada; c Services et Recherches Psychiatriques AD, Montreal, Canada; d Institut national de Psychiatrie Légale Philippe-Pinel, Montreal, Canada

**Keywords:** COVID-19, virtual reality therapy, cognitive behavioural therapy, schizophrenia, avatar therapy

## Abstract

**Purpose:**

The COVID-19 pandemic led to exacerbation of mental health symptoms and deterioration in psychological well-being in individuals suffering from schizophrenia. The primary objective of this study is to evaluate the impacts of the COVID-19 pandemic on patients suffering from treatment-resistant schizophrenia (TRS) with auditory verbal hallucinations (AVH) having undergone virtual reality therapy (VRT) or cognitive behavioural therapy (CBT) on their symptomatology. The secondary objective is to identify the differences and similarities in relation to the response to the COVID 19 pandemic between these two groups of patients.

**Methods:**

Qualitative analysis of semi-structured interviews was conducted with 42 patients suffering from TRS who had previously followed VRT or CBT. All interviews were recorded, transcribed, and analysed.

**Results:**

Four themes emerged in this study: Psychotherapeutic Interventions, Impact of COVID-19 and Public health and safety policies, Substance use and Psychiatric follow-up. Participants from both groups reported that their therapy was beneficial in controlling AVH. Patients having followed CBT reported more depressive symptoms whereas patients having followed VRT reported more anxious symptoms.

**Conclusions:**

This study offers a first qualitative insight in patients suffering from TRS and the impacts of COVID-19 on them and opens the door to the protective factors of CBT and VRT for this specific population.

## 1. Introduction

Schizophrenia is a severe mental illness characterized by the presence of delusional beliefs, hallucinations, as well as disturbances in thought, perception and behaviour [1]. Around 30% of patients suffering from schizophrenia do not respond to two or more trials of antipsychotics and this is known as *treatment-resistant schizophrenia* (TRS) [2]. TRS is a complex, severe and disabling psychiatric disorder, which poses a significant therapeutic challenge and current treatments show limited efficacy [2]. Reduced access to healthcare can be a risk factor for psychotic events and relapses [3].

The COVID-19 pandemic has disrupted the daily lives and has had tremendous impacts on the psychological well-being of countless people [4]. Individuals suffering from pre-existing mental disorders have been especially affected. Indeed, the pandemic has been associated with an exacerbation of psychological symptoms, including anxiety, depression and post-traumatic symptoms [5]. This exacerbation of mental health symptoms and deterioration in psychological well-being of individuals suffering from pre-existing mental disorders was more specifically observed in patients suffering from schizophrenia [6]. To prevent the spread of the virus, many measures have had to be put in place, which has led to decreased access to mental health services and reduced psychiatric consultations and care for psychiatric patients to prevent the spread of the virus have been described [7]. Moreover, fear of COVID-19 exposure, and restrictions in public transport have led to difficult access to medication and reduced care seeking [8]. Due to the lack of mental health resources since the beginning of the pandemic, it is likely that patients’ psychological well-being has been more at risk. Patient suffering from schizophrenia have seen a greater risk of adverse outcomes from COVID-19 [9].

An exacerbation of psychotic symptoms due to the pandemic was found in these patients. For example, public health measures, such as people wearing face masks, have led to increased paranoia in patients suffering from schizophrenia [10]. Moreover, individuals may experience auditory hallucinations in relation to the coronavirus and incorporate the pandemic narrative in their delusional world [11]. Some individuals with schizophrenia developed intrusive and paranoid thoughts about the virus [10]. Patients suffering from schizophrenia are also more prone to fear being infected, developing obsessive thoughts about the illness blaming oneself for the pandemic, or even to self-harm showing that the pandemic can trigger severe behavioural alterations [12]. An increase in anxiety and depressive symptoms was also observed in patients with schizophrenia [13].

Up to 50% of patients suffering from schizophrenia are resistant to first-line antipsychotic treatments and will continue to suffer from persistent auditory verbal hallucinations (AVH) [14]. These patients can benefit from additional non-pharmaceutical approaches such as psychotherapeutic approaches. Cognitive behavioural therapy (CBT) can be applied in combination to medication for these patients [15]. Several studies have outlined that CBT can be helpful in the reduction of emotional distress associated with auditory hallucinations for these patients. However, at best only moderate effects are achieved [16]. This has led to the need for the development of new psychosocial interventions for the treatment of TRS. These new interventions can focus on processes specific to voice hearing and include experiential elements within the therapy [17]. One new therapy that has been developed for TRS is virtual reality (VR) integrated within psychotherapy. The use of VR is especially useful in the case of schizophrenia for the treatment of AVH since simple exposure is challenging due to the invisible component of voices. It is consequently possible to enhance current psychological approaches by allowing patients to be directly exposed in a personalized manner to their anxiety-inducing voices *via* VR [18]. VR-assisted therapy (VRT) is experiential and uses exposure-based interventions that permit the establishment of an intimate dialogue with patients’ voice by means of an avatar controlled in real time by a therapist. The results of the two pilot trials comparing VRT to treatment-as-usual as well as a larger RCT comparing Avatar Therapy, a form of VRT, to supportive counselling demonstrated large effects of VR therapies on AVH in short-term follow-ups and up to a 24-week follow-up [19,20]. However, the COVID-19 pandemic limited access to these modalities for these patients. It was highlighted that some patients were less compliant to their care because of the reduced availability for psychotherapeutic approaches or because of the change in modalities (i.e. from in-person therapy to telemedicine) [21].

There is, to our knowledge, no literature that assesses the potential protective effects of having been exposed to CBT or VRT for patients suffering from TRS. While there is a growing body of evidence highlighting the effects of VRT on AVH and quality of life, among others, and the effects of CBT for treatment of TRS, this study seeks to gather data on the potential protective factors of VRT or CBT on TRS patients facing the COVID-19 pandemic. While there is no direct literature assessing the protective factors inherent to therapeutic modalities in the situation of a pandemic, certain therapies, such as trauma-focused therapy has demonstrated that it can protect from relapse over time [22]. Observations about how patients having followed VR or CBT lived through the pandemic could allow further evaluation of these therapies for TRS.

The main objective of this content analysis study is to evaluate the positive and negative impacts of the COVID-19 pandemic on TRS patients having undergone VRT or CBT on their symptomatology, their daily life, and their usual psychiatric follow-up. Secondary objectives were to identify the differences and similarities in relation to the response to the COVID-19 pandemic between patients who followed VRT and those who followed CBT, as well as the differences and similarities in relation to the response to the COVID-19 pandemic between patients who responded well to VRT and patients who did not respond well to VRT. We hypothesize that patients having undergone VRT or CBT will have had stability in their symptomatology and their daily life while their usual psychiatric follow-up might have been impacted because of external factors linked to the COVID-19 pandemic.

## 2. Materials and methods

### 2.1. Participants

The participants in this study were selected from two completed pilot trials at the *Institut universitaire en santé mentale de Montréal* (IUSMM) and one ongoing trial (2017–2021) [20,23]. To be included in the pilot trials, participants needed to be 18 years old or older, had received a diagnosis of schizophrenia or schizoaffective disorder with persistent AVH and must have failed to respond to two or more antipsychotic trials. Participants were excluded from the trial if they had a neurological disorder, a severe physical illness, or a substance use disorder in the past year. These patients were all suffering from TRS and were randomized in either CBT or VRT during their participation in the above clinical trials. Out of the 60 potentially eligible participants (30 who received VRT, 30 who received CBT), 15 refused to participate in this study (8 in the VRT group, 7 in the CBT group), 2 were unreachable (2 in the VRT group) and 1 patient was deceased (who received CBT) for a total of 42 participants.

### 2.2. Sampling and recruitment

The participants, who were originally referred from the IUSMM or the community and had previously been randomized in the former studies between VRT and CBT were recruited for this study. VRT in the above-mentioned trials consisted of 9 weekly 1 h sessions in which patients are immersed using a virtual reality headset to confront a visual representation (avatar) of their most distressing voice. The first session consisted of creating the avatar, sessions 2 and 4 are designed to confront the patients to their hallucinatory experience, whereas session 5 is designed to target self-esteem and the remaining sessions are consolidating sessions. The CBT intervention consisted of 9 weekly 1 h session of manualized CBT therapy of learning modules and tasks to complete. Further details about the ongoing trial and the methodology of the VRT and CBT interventions can be found in Dellazizzo et al. [20]. All patients who had participated in the three VRT/CBT pilot trials were called by phone by NL and KSS during the lockdown period in the summer of 2020. They were asked about their interest to participate in a follow-up interview in relation to their previous experience with VRT or CBT, and to their experience of the pandemic and lockdown so far. If participants consented to the open-ended interview, an interview guideline containing open-ended questions was asked to all participants during the same phone call. Study has been approved by the ethics committee of the *Centre de recherche de l’IUSMM* (CR-IUSMM).

### 2.3. Data collection

Semi-structured interviews were conducted by medical students NL and KSS and cross-validated by AH, a graduate student and lasted approximately 30 min each. The interviews questions were formulated by assessing the impact COVID-19 pandemic on the major themes that are assessed during the VRT and CBT sessions. The following topics were included in the open-ended questionnaire to structure the interviews:

- The impact of the COVID-19 pandemic on the participants psychotic symptoms (changes in AVH).
- The impact of the COVID-19 pandemic on the participants daily lives, activities, general wellbeing, and social interactions.
- The impact of the COVID-19 pandemic on the participants affective symptoms (depressive or anxious), including suicidal thoughts.
- The participant’s psychiatric and psychological follow-up during the COVID-19 pandemic.
- The participant’s impression and personal experience related to the therapy they had previously followed (VRT or CBT).

All interviews were recorded and transcribed in full by NL and KSS. A total of 42 transcripts were obtained. Interview questions provided a framework for discussion, while the interview format allowed participants to explore issues not anticipated by the questionnaire, therefore, allowing for the possibility of producing unexpected findings.

### 2.4. Data analysis

Following transcription, emerging themes were individually extracted in 42 transcripts following the design framework of grounded theory [24]. Second, a preliminary coding scheme, from these emerging themes, was constructed by NL and KSS and was used to annotate five transcripts using *Qualitative Data Analysis Miner* software [25]. Third, annotations conducted by NL and KSS were compared: the interrater agreement of the coding of the verbatims was compared using the Scott’s Pi. A Scott’s Pi is a measure of the interrater agreement (degree of agreement among raters, a score of how much homogeneity or consensus exists in the ratings given by various judges) [26]. The list and definition of themes were updated in relation to the differences found between the two raters coding all verbatims, by dividing themes, grouping codes into themes, adding new themes, or removing others. With this updated list and definition of themes, the raters coded five new randomly selected interviews with QDA miner. This process was repeated until the Scott’s Pi obtained was acceptable and saturation of data was achieved. Acceptability was defined as per the benchmark provided by the SAGE Research Methods in which a Scott’s Pi of 0.81–1.00 is indicative of an almost perfect agreement, 0.61–0.80 of a substantial agreement, 0.41–0.60 of a moderate agreement, 0.21–0.40 of a fair agreement, 0.0–0.20 of a slight agreement and less than 0 as a poor agreement [26]. During the first iteration of the themes, the authors had a Pi score of 0.52 with 24 themes and 5 transcripts. After changes to the themes, the score was 0.65 with 19 themes. A total of 10 verbatims were assessed. In the last iteration of their annotation, NL and KSS reached a Pi score of 0.77 with modification on the definitions of the themes.

## 3. Results

### 3.1. Sample characteristics

A total of 42 participants were interviewed; 20 participants had received VRT as part of their treatment whereas 22 had received CBT. All participants had a diagnosis of TRS. Sociodemographic characteristics as well as *The Psychotic Symptom Rating Scales* (PSYRATS) auditory hallucination score and the Positive and Negative Syndrome Scale (PANSS) for anxi-depressive symptoms for both groups can be found in Table 1. Patients who refused to participate in the study in the VRT group as compared to the ones from the CBT group had no statistical difference in their sociodemographic characteristics and symptoms severity.

**Table 1.** Sociodemographic characteristics and symptoms per intervention.

	CBT	VRT	*p* Value
Sex (male, female)	17,5	15, 5	.213
Age (mean in years)	43.2 ± 11.6	41.1 ± 11.0	.178
Education (mean in years)	13.2 ± 4.2	13.6 ± 4.6	.630
Ethnicity (Caucasian, visible minorities)	90.1%, 9.9%	90%, 10%	.959
Average time since last therapy session (mean in months)	15.1 ± 2.6	14.8 ± 2.1	.372
% on Clozapine	40.0	36.4	.470
PSYRATS-AH-Total (mean score)	29.30 ± 6.0	29.52 ± 4.2	.739
PANSS-Anxio-depressive (mean score)	9.00 ± 2.6	10.10 ± 2.4	.588

Four major themes that have emerged from the interviews and their sub-themes may be found in Table 2. The four themes have been divided into 18 sub-themes in which all the interactions from the interviews were classified.

**Table 2.** Themes and sub-themes identified.

Theme	Sub-theme	Verbatim (example)
Psychotherapeutic interventions (CBT or VRT)	Opinion (positive or negative) of the impact of the therapy regarding the pandemic on the everyday life of the patient.	“I am under the impression that VRT helped me fight my voices during this dark period”.
	Impact of the therapy regarding the pandemic on the intensity and frequency of the AVH.	“I think VRT has helped me reduce the number of times the voices annoy me during the day”.
	Impact of the therapy regarding strategies to control AVH	“I try to tell him to go away like I did during VRT”.
Impact of COVID-19 and public health safety policies	Impact of COVID-19 related to their ability to take care of themselves	“COVID-19 ruined my social circle. I feel isolated”.
	Impact of COVID-19 related to their work	“I did not work prior to COVID-19, so it did not change anything”.
	Impact of COVID-19 related to their social network	“My family could not visit me during the pandemic”.
	Impact of COVID-19 related to their finances	“COVID-19 did not impact my finances”.
	Impact of COVID-19 related to their everyday activities	“I couldn’t go volunteer because of that pandemic”
	Impact of COVID-19 related to their personal projects, endeavours	“The pandemic did not stop me from doing my usual stuff”.
	Impression of the global impact of the pandemic on their well-being	“COVID-19 did not affect my well-being”.'
	Opinion on the public and health safety policies	“'I do not like the fact that there are so many rules, but I follow them”.
	Opinion on the impact of the pandemic on their AVH	“My voices are pretty much the same since the pandemic. Nothing has changed”.
	Opinion on the impact of the pandemic on their mental state regarding their mood, anxiety, and psychotic symptoms.	“I feel much more anxious since this virus thing is around”.
Substance use	The type of substance they use.	“I smoke cigarettes everyday as usual”.
	Changes in their substance usage.	“I drink two beers everyday like I did before”.
Psychiatric follow-up	Changes in their follow-up	“I have been in contact with my counsellor every day by phone since the beginning of the pandemic, this is the same as before”.
	Changes in medication, medical condition, or global health.	''My health is the same as before. ''
	Opinion on how the pandemic affected their psychiatric follow-up.	“Some of my appointments were by phone instead of in-person because of the virus”.

#### 3.2.1. First theme: psychotherapeutic interventions

This theme encompasses three sub-themes that assess the impact of the psychotherapeutic process (CBT or VRT) regarding how they cope with the COVID-19 pandemic.

Regardless of the type of therapy the participants followed, most participants reported that they were beneficial in keeping control on their AVH. About 72.7% of the CBT group reported a positive impact of the therapy regarding the pandemic on the everyday life as compared to 80% of the VRT group. Participants reported in most of the verbatims that they believe their therapy helped them overcome their AVH on their day-to-day basis despite the COVID-19 pandemic. Meaningful units of text supported this such as “I believe that what I learned in CBT is still useful for me to handle my voices” Similar units were identified for the VRT group such as “I sometimes remember how I used to respond to my avatar to control my voices, I am still the one in charge”.

Of the 22 participants having previously followed CBT, 13.6% experienced an increase in frequency of their AVH, 72.7% experienced no changes, and 13.6% noticed a decrease in their AVH since the beginning of the pandemic. Therefore, across all participants, there is a global tendency in both groups in stability of AVH frequency and intensity, despite the pandemic. The participants who reported the increase in frequency of their AVH reported that the pandemic was stressful and had a direct impact on the voices they are hearing. One patient stated that he feels that despite CBT provided him with a better understanding of why he is hearing voices; he was not able to make them go away by distracting himself like he did before. Similar statements emerged from this group. Of the participants who mentioned a decrease in their AVH, statements such as “(…) the pandemic is very difficult for me, I believe, however, that having followed CBT gave me the strength to keep on working every day to make the voices annoy me less frequently (…)” were common.

From the 20 participants having previously followed VRT, 20% noticed an increase in frequency of their AVH, 60% noticed no changes and 20% noticed a decrease in their AVH since the beginning of the pandemic. From these participants, the ones that experienced an increase in frequency reported that they had difficulties referring to how they interacted with their avatar and apply this in the context of the pandemic because the stress was “(…) just so intense”. The participants who reported a decrease in their AVH stated that VRT provided them with the necessary confidence to keep their control on their AVH and have the strength to repel them.

Both groups reported that there was no change in the intensity of their AVH. Statements such as “(…) I believe my therapy helped me and my situation with the voices did not change since the pandemic” were frequent.

#### 3.2.2 Second theme: impact of COVID-19 and public health safety policies

This theme includes 10 sub-themes that assess the direct or indirect impact of COVID-19 and the related public health safety policies. It includes their impact on the different functional areas of the participants and their impression of the public health safety policies, the impact of the pandemic on their AVH and their mental state.

In both groups, impact on work, social network, ability to take care of themselves and finances were not reported as influenced by the pandemic by the participants. Statements such as “(…) my situation has not changed since the pandemic” were frequent when inquiring about these different areas. Participants experienced frustrations regarding the public safety policies in both groups by mentioning the mask and the sanitary measures were annoying. However, in both groups, they argued that such measures were probably necessary if everyone was doing them: “In my building, it is mandatory to wear the mask when I walk in the hallways. It is not so strange considering everyone is doing it but I am not sure if it is very helpful.” Other participants stated that there were too many rules, but they were going to follow them regardless: “Every day they come up with something new! Are they going to make up their mind”? None of the participants reported opposed themselves to the sanitary measures. Regarding the social networks, most participants reported that they were isolated but still were able to reach their family and friends by phone as they did prior to the pandemic.

Twenty percent of the participants having followed VRT discussed the presence of depressive symptoms in direct relation to the pandemic in their interview, compared to 31.8% of the group having followed CBT. Feelings of sadness, emptiness and voidness were reported by these participants. Statements such as “Being isolated from my friends is making me sad” were identified for most of these participants. Depressive symptoms in relations to the impact of COVID-19 have not been mentioned by the other participants. Therefore, more patients having followed CBT expressed depressive symptoms in direct relation to the pandemic compared to patients having followed VRT.

The presence of suicidal ideation since the pandemic was outlined by 20% of the participants having followed VRT and 36.4% of the participants having followed CBT. Units of text highlighting suicidal ideation did not include any sort of suicide planification or attempts and were mostly linked to de-valorization. Passive suicidal ideation such as “I would be better off dead, my life is worth nothing” were the most common. It is unknown if participants reported these suicidal ideation to their care providers.

A total of 65% participants having followed VRT discussed the presence of anxiety symptoms attributed to the pandemic in their interview, compared to 31.8% of the participants having followed CBT. The remaining participants did not report symptoms related to anxiety. Statements about anxiety included units of text such as: “I feel stressed because the pandemic is something we cannot control,” “The COVID-19 pandemic has been nerve-racking, there are so many people dying right now” and “I imagine the worst when I see all these people wearing masks, are we going to survive this”?

#### 3.2.3. Third theme: substance use

This theme, divided in two sub-themes, refers to the participants current consumption habits regarding alcohol and drug use. It encompasses the type of intake and the changes in consumption habits since the COVID-19 pandemic.

None of the participants in both groups reported a change in their consumption habits since the COVID-19 pandemic. Statements for both groups commonly referred to their consumption habits as identical as prior to the COVID-19 pandemic. Units of text such as “Nothing changed” were the most common. Participants in both groups mentioned that they still were able to purchase their cigarettes and liquors without any problems: “(…) the convenience store was still open so it was not difficult for me to buy my cigarettes.” Two participants from the CBT group mentioned that peer helpers brought them their groceries (including alcohol and cigarettes). Participants rapidly overlooked this section of the semi-structured interview by dismissing the importance of their consumption habits in their daily life. Participants who were using substances such as cannabis, amphetamines, cocaine, and other psychoactive substances did not report any changes in how they procured themselves these drugs.

#### 3.2.4. Fourth theme: psychiatric follow-up

This theme encompasses three sub-themes that assess the nature of the psychiatric follow-up of the participants, any biological changes (i.e. change of medication or medical condition) and their general opinion on their follow-up during the pandemic.

In relation to their current psychiatric follow-up since the pandemic, participants had a general positive feeling of the follow-up, this theme being expressed by 72.7% of the participants having followed CBT and 75% of the participants having followed VRT. Participants reported that they were happy overall with their follow-up. One patient of the CBT group mentioned that he felt his treating team was going out of their way to ensure he was well treated. The remaining participants in both groups expressed a negative feeling about their psychiatric follow-up, mainly because of the reduced number of availabilities by their care providers. Statements such as “(…) my psychiatrist stopped seeing patients in person (…)” emerged in this group. Several participants reported stability in their medication since the pandemic (54.5% CBT, 45% VRT), and few participants described an increase or decrease in their medication during that same period (13.6% CBT, 10% VRT). Strategies such as calling their care centres or calling help hotlines were the most used by the participants when they felt they needed assistance.

## 4. Discussion

The first objective of this study was to evaluate the impacts of the COVID-19 pandemic on TRS patients having undergone VRT or CBT and second to identify differences and similarities amongst them. Four major themes emerged in this study: Psychotherapeutic Interventions, Impact of COVID-19 and Public health and safety policies, Substance use and Psychiatric follow-up. Important findings of this study are that AVH were reported as stable in both groups, less depressive symptoms were reported in the VRT group and less anxiety symptoms were reported in the CBT group.

All the participants in this study expressed a general stability in their AVH regardless of the psychotherapeutic intervention they had received. Several studies support that VRT can have a long-term positive impact on the stability of AVH [27]. Long-term improvements in positive symptoms of schizophrenia are also reported for patients having followed a full treatment of CBT in a trial from 2010 consisting of 33 patients suffering from schizophrenia [28]. Our results are consistent with these findings and may hint that despite significant events such as a pandemic, having followed VRT or CBT for patients suffering from TRS might be a protective factor considering both groups of patients presented stability in their AVH.

With regards to the impact of the COVID-19 and the public health and safety policies, findings were similar for both groups except for anxiety and depressive symptoms which are comparable to other studies. For example, the COVID-19 pandemic has been reported to increase anxiety and depressive symptoms amongst patients with mental illness [29]. A study examining 2734 psychiatric patients reported worsening of psychiatric conditions in two thirds of the patients assessed [30]. This has been reportedly the case for patients suffering from psychotic disorders such as schizophrenia mainly because of their lack of self-care ability and neurocognitive impairment [31]. Contrary to previous research, participants in this study reported to a generally stable state of their AVH. Several studies outline the moderate benefit of CBT in the reduction of AVH in patients with psychotic disorders. A study from Peters et al. [32] who assessed the impact of CBT on AVH 12 months after the therapeutic sessions came to the same conclusions [32]. A larger number of participants having followed CBT reported depressive symptoms. In the VRT group, more participants having followed this therapeutic modality reported anxious symptoms. It can be hypothesized that as CBT and VRT are both distinct therapies following different targets and therefore they might have divergent effects. CBT is a structured, time-limited, problem-focused and goal-oriented form of psychotherapy that brings the patient to identify, reflect and change how they behave [33]. When assessing for VRT, it is a novel therapy in which people who hear voices have a dialogue with an avatar of their AVH, voiced by the therapist so that the avatar responds by becoming less hostile and concedes power over the course of therapy [34]. Therefore, it could be hypothesized that participants having followed VRT are less inclined to depressive symptoms as they acquired a more engaged role in their control of their AVH and maybe better control of stressful situations in their life. Similarly, avatars are being studied in the treatment of patients suffering from depression and a growing body of evidence support their use [35]. CBT, targets cognitive processes which might have helped participants in having a broader comprehension of their hallucinations and the anxiety might be purely derived from the new external factor: the pandemic. There is a lack of data about VRT on its impact on the cognitive processes. It is to be noted that while differences were noted in the interviews, most of the participants did not report anxiety or depressive symptoms, which may reflect that most patients benefitted from both therapies and manage their emotions adequately during stressful events. This could be explained that hallucinations can be noted as stressful events in the daily life of patients and, as mentioned previously, VRT and CBT trials hint that these approaches are preparing patients to face stressful events.

None of the participants in both groups presented a change in their substance use habits. While pre-existing substance-use can be linked to health issues related to being infected with COVID-19, the pandemic itself in relation to substance use in patients suffering from TRS is limited. However, few studies indicate an increase in substance use in the general population during the pandemic [36,37]. One hypothesis could be that participants from this study were exposed to a general lockdown of the entire province during the peak of the pandemic, therefore limiting possible access to substances.

Considering their psychiatric follow-ups, most participants did not report an impact in their accessibility to their usual care regimen. However, in both groups, a small amount reported a decrease in accessibility. Studies emerging since the COVID-19 pandemic have outlined the importance of an integrated collaborative care approach for patients suffering from mental illnesses [38]. Participants from this study already had a care team in place prior to the pandemic which might explain why most of the participants had stability in their usual care. Lack of accessibility to health care has been described worldwide in relation to the pandemic which might explain why some participants from this study highlighted a breach in access to their usual care.

## 5. Limitations

This remains an exploratory study. The lack of generalisation of results to other interventions limits its interpretation and external validity. This study was done retrospectively and can be bound to re-call bias. Considering that cognitive impairment is frequent in the population of patients suffering from schizophrenia, participants could have forgotten important piece of information or omitted pertinent details. Semi-structured interviews, while flexible, can limit reliability. Finally, this study did not include a control group that did not follow one of the two therapies. Therefore, there was a lack of objective data to include potential comparison groups such as the non-participants in the VRT and CBT trials and those who did not accept to participate in the interview, which could have enabled a comparison with patients suffering from schizophrenia following treatment as usual.

## 6. Conclusions

In conclusion, the objective of this study was to evaluate the impacts of the COVID-19 pandemic on TRS patients having undergone VRT or CBT and to note differences and similarities amongst them. Using content analysis, four key themes were defined and were subdivided into 18 sub-themes. It has been reported that both groups of participants have AVH that are stable in frequency and intensity. A small body of participants who undergone CBT reported more depressive symptoms. It was observed in the VRT group that few participants reported anxiety symptoms. Fortunately, both groups of participants reported stability in their psychiatric follow-up and plan of treatment. VRT and CBT could therefore be important avenues to chronically improve AVH in TRS patients despite external stressful situations such as pandemics. While this study offers a first qualitative insight in patients suffering from TRS and the impacts of COVID-19 on them, it opens the door to the protective factors of CBT and VRT for this specific population.

## Registration number

Patients that are part of this study were selected based on the proof-of-concept trial from Percy du Sert 2018 ‘s study and Dellazizzo 2021s study [20,23]. This clinical trial has been registered on Clinicaltrials.gov (identifier number: NCT03585127).

## Ethics institutional review board statement

This study was approved by the institutional ethical committee, and written informed consent was obtained from all patients. Patients that are part of this study were selected based on the proof-of-concept trial from Percy du Sert 2018 ‘s study and Dellazizzo 2021s study [20,23]. The trial was conducted in accordance with the Declaration of Helsinki and was approved by the institutional ethical committee (CER IPPM 16-17-06). We obtained written informed consent from all patients.

## Informed consent statement

Informed consent was obtained from all subjects involved in the study.

## Author contributions

All the authors meet the ICMJE definition of authorship. A.H. and A.D. designed the study, carried out the analysis and interpretation of data, wrote the article and revised critically the article. N.L. and K.S.S participated in data acquisition, carried out the analysis and revised critically the article. K.P. recruited the cohort, participated in data acquisition analysis and revised critically the article. L.D. participated in the design of the study and revised critically the article. All authors have read, agreed to the published version of the manuscript and are accountable for all aspects of the work.

## Data Availability

The data presented in this study are available on request from the corresponding author. The data are not publicly available due to patients’ privacy.
